# Brain metastasis in stage IV lung adenocarcinoma is frequently missed by symptom-based screening

**DOI:** 10.1007/s12672-025-04210-7

**Published:** 2025-12-11

**Authors:** Sama I. Sayin, Ella A. Eklund, Moa Beischer, Torill Moe, Kevin X. Ali, Kerstin Gunnarsson, Moe Xylander, Lars Ny, Asgeir S. Jakola, Ida Häggström, Clotilde Wiel, Andreas Hallqvist, Volkan I. Sayin

**Affiliations:** 1 Sahlgrenska Center for Cancer Research, Department of Surgery, Institute of Clinical Sciences, University of Gothenburg, Gothenburg, Sweden; 2 Wallenberg Centre for Molecular and Translational Medicine, University of Gothenburg, Gothenburg, Sweden; 3 Department of Oncology, Sahlgrenska University Hospital, Gothenburg, Sweden; 4 Department of Oncology, Institute of Clinical Sciences, University of Gothenburg, Gothenburg, Sweden; 5 Department of Neurosurgery, Sahlgrenska University Hospital, Gothenburg, Sweden; 6 Institute of Neuroscience and Physiology, Section of Clinical Neuroscience, University of Gothenburg, Gothenburg, Sweden; 7 Department of Electrical Engineering, Chalmers University of Technology, Gothenburg, Sweden; 8 Department of Medical Radiation Sciences, University of Gothenburg, Gothenburg, Sweden

**Keywords:** Brain metastasis, Diagnostic brain imaging, Lung adenocarcinoma, Neurological symptoms, Screening, Real-world data

## Abstract

**Background:**

Brain metastases (BM) are a major clinical challenge in metastatic lung adenocarcinoma (LUAD), affecting up to 50% of patients during disease progression. Current guidelines do not mandate brain imaging for all metastatic lung cancer patients at diagnosis unless there are neurological symptoms present. However, real-world data on the predictive value of neurological symptoms for BM detection remain scarce.

**Methods:**

This retrospective multicenter study analyzed all consecutive patients diagnosed with stage IV LUAD with molecular assessment in western Sweden from 2016 to 2021 (*n* = 912). We extracted data from patient charts, imaging referrals, radiology reports and the Swedish National Lung Cancer Registry to determine diagnostic brain imaging (DBI) frequency and modality, presence of neurological symptoms, BM detection rates, size, number, location and overall survival (OS).

**Results:**

Among stage IV LUAD patients, 63% underwent DBI, and BM was detected in 23% of all patients (37% of those receiving DBI). Neurological symptoms prompted DBI in 63% of cases, yet 58% of these symptomatic patients had no BM on imaging. Conversely, 28% of asymptomatic patients who underwent DBI had BM. Patients with BM detected in the absence of neurological symptoms had smaller metastases. Neurological symptoms were associated with worse OS, irrespective of the presence of BM.

**Conclusion:**

Neurological symptoms alone do not reliably predict the presence of brain metastases in stage IV LUAD. In this real-world cohort, symptom-triggered imaging was associated with under-detection of asymptomatic BM. Our findings support the need to re-evaluate current symptom-based screening practices and may inform future efforts toward more standardized brain imaging strategies in metastatic NSCLC.

**Supplementary Information:**

The online version contains supplementary material available at 10.1007/s12672-025-04210-7.

## Introduction

Lung cancer remains the leading cause of cancer-related mortality worldwide, with advanced-stage patients facing a five-year survival rate below 10%, highlighting the urgent need for optimized clinical management. In Sweden, lung cancer is the fifth most common type of cancer but still accounts for the highest number of cancer-related deaths each year [1].

Lung adenocarcinoma (LUAD), the most common subtype of non-small cell lung cancer (NSCLC) and accounting for 50–60% of all lung cancer cases, poses significant clinical challenges, particularly when it metastasizes to the brain [2, 3]. Brain metastases (BM) occur in 25–30% of LUAD at diagnosis, and up to 50% of LUAD will develop BM during disease progression [4–6]. BM significantly worsens prognosis and often leads to debilitating neurological symptoms, impacting quality of life and requiring intervention of metastatic lesions in this organ to a larger extent compared to patients with metastases in other organ sites [7–10].

While targeted therapy and immunotherapy are paving the way for novel approaches to treating BM, neurosurgical resection and stereotactic radiosurgery remain the most effective tools we have to date, and outcomes following both are adversely impacted by increasing tumor size and numbers [11]. Early detection of BM might improve outcomes, enabling timely multimodal treatment, particularly as the number of long-term BM survivors continues to rise together with improved treatment outcomes.

Clinical practice and international guidelines agree that presence of neurological symptoms at diagnosis of Stage IV LUAD should prompt screening brain imaging, with MRI preferred over CT for its superior sensitivity and staging utility [12–21]. At the same time, contemporary cohorts, systematic reviews, and real-world studies show that a substantial proportion of BM in NSCLC are asymptomatic at detection, and that MRI frequently reveals lesions missed on CT – meaning a symptoms-only approach underestimates intracranial disease [4, 6, 15, 22–25]. In parallel, multicenter audits, health-system analyses, and observational cohorts indicate that performing baseline MRI in advanced disease identifies otherwise occult BM, influences initial management (local therapy planning, systemic regimen selection, trial eligibility), and in some settings is associated with signals toward improved outcomes, particularly in fitter patients [15, 22, 23, 26–30]. Yet, current guidelines mandate imaging only when symptoms are present and otherwise leave baseline screening to the discretion of the clinician.

To map real-world outcomes amid the current tension between guidelines and the scientific evidence, we performed a retrospective, multicenter cohort study of all consecutive patients with metastatic LUAD who underwent molecular assessment in western Sweden (2016–2021), integrating data from patient charts, imaging referrals, radiology reports, and the Swedish National Lung Cancer Registry. Our objectives were to characterize current real-world DBI practice, document neurological symptom status at the time of imaging, and quantify how well symptoms predict the presence of brain metastases. We also compared detection by modality (CT vs. MRI), including CT-negative/MRI-positive cases, to assess the performance and clinical implications of a symptom-based screening approach in routine care.

## Materials and methods

This study provides a real-world overview of how DBI was utilized in the clinical management of patients diagnosed with metastatic LUAD in western Sweden between 2016 and 2021. We conducted a multicenter retrospective study by combining data from the Swedish National Lung Cancer Registry with data from patient charts, imaging referrals and radiological reports for each patient. The Swedish national guidelines for lung cancer are in line with ESMO guidelines. By combining data about the frequency of DBI and whether the imaging was done due to neurological symptoms or not, we investigate whether symptoms is a good indicator for DBI. The Swedish healthcare system is primarily government-funded and provides universal access to all citizens. Therefore, all patients have equal access to diagnostic examinations and treatments.

### Patient population

**Fig. 1:**
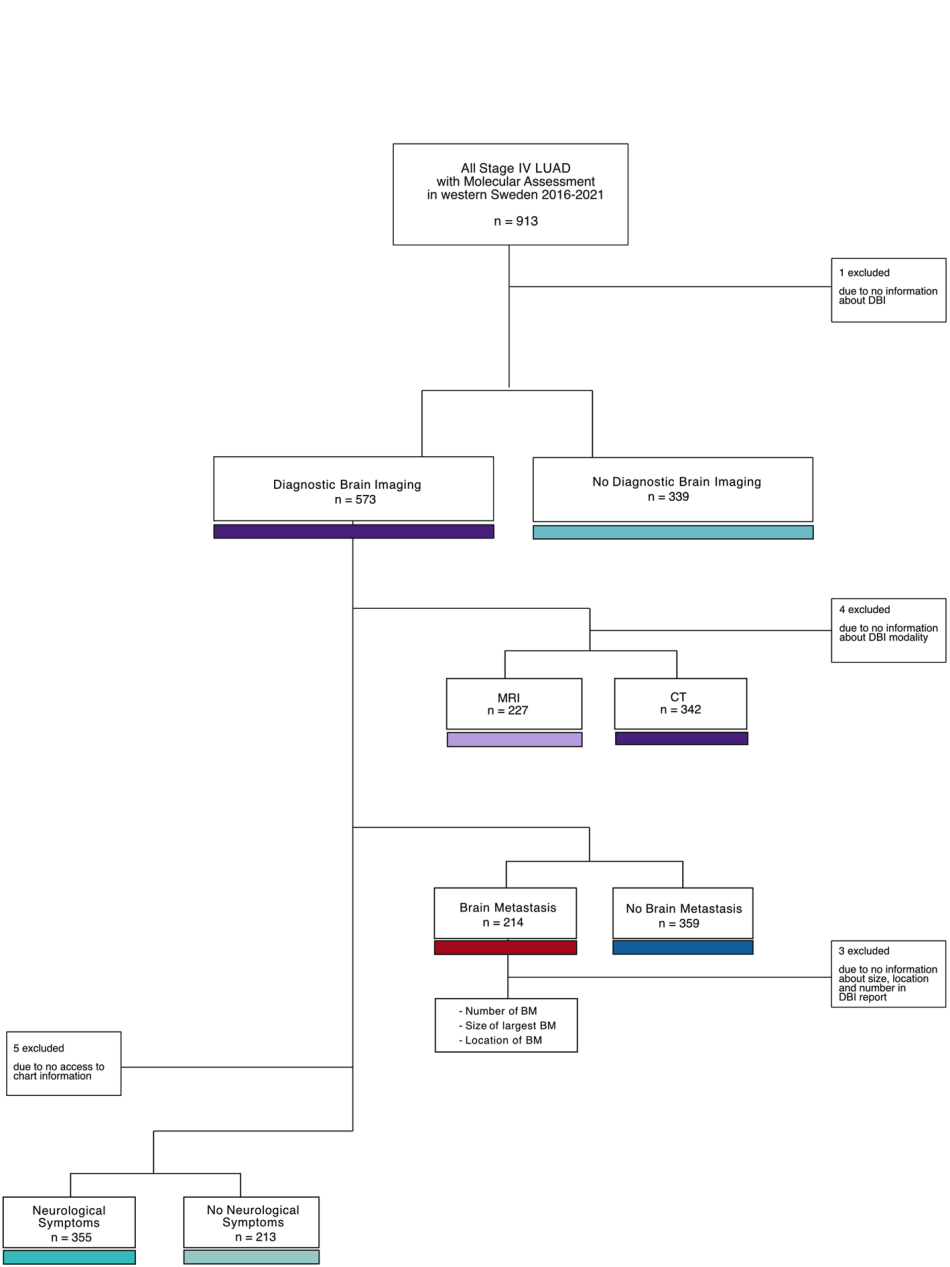
Flowchart showing patient selection for the study

We included all consecutive lung cancer patients diagnosed with Stage IV LUAD and having molecular assessment performed between 2016 and 2021 in western Sweden (*n* = 912). One patient was excluded due to no DBI information being available, and one patient died between diagnostic sample collection and final diagnosis and is thus excluded from the overall survival (OS) analysis. Five patients included in the Swedish National Lung Cancer Registry did not have an available chart to investigate the presence of neurological symptoms and were therefore excluded from the analyses that used that information (Fig. 1). Four patients did not have DBI modality reported, and three DBI reports of BM patients did not contain details of tumor size, location and diameter. One patient from the CT group was excluded from imaging analyses since the CT was performed without contrast. One patient from the MRI group was excluded from survival analysis since survival data was not available.

Patient demographics (including age, gender, Eastern Cooperative Oncology Group (ECOG) performance status and smoking history) and outcome data were retrospectively collected (Table 1).

All patients were treatment-naïve at the time of data collection and had confirmed stage IV disease at diagnosis. For patients with no contraindication to contrast, brain imaging was typically performed using contrast-enhanced CT, which was the standard approach in Sweden during the study period. MRI was used at the clinician’s discretion, typically in cases with inconclusive CT or when clinical symptoms warranted higher-resolution imaging.

All patient charts were examined for whether a DBI was conducted or not. If DBI was done, the chart and imaging referral were examined to identify if it was done due to neurological symptoms being present or not. Neurological symptoms were defined as signs and symptoms that were deemed to be associated with BM according to clinical judgement, and included headaches, seizures, vertigo, balance disturbances, changes in cognition, motor or sensory deficits and visual or speech disturbances, as specified in Table 2. If the DBI detected brain metastasis, the number, location and largest diameter of metastases were collected from the DBI report. CT was considered to be positive for BM if DBI report by radiologist explicitly confirmed presence of BM or if malignancy was suspected. Approval from the Swedish Ethical Review Authority (Dnr 2019–04771 and 2021–04987) was obtained prior to the commencement of the study. No informed consent was required due to all data being presented in a de-identified form according to the Swedish Ethical Review Authority.

**Table 1:**
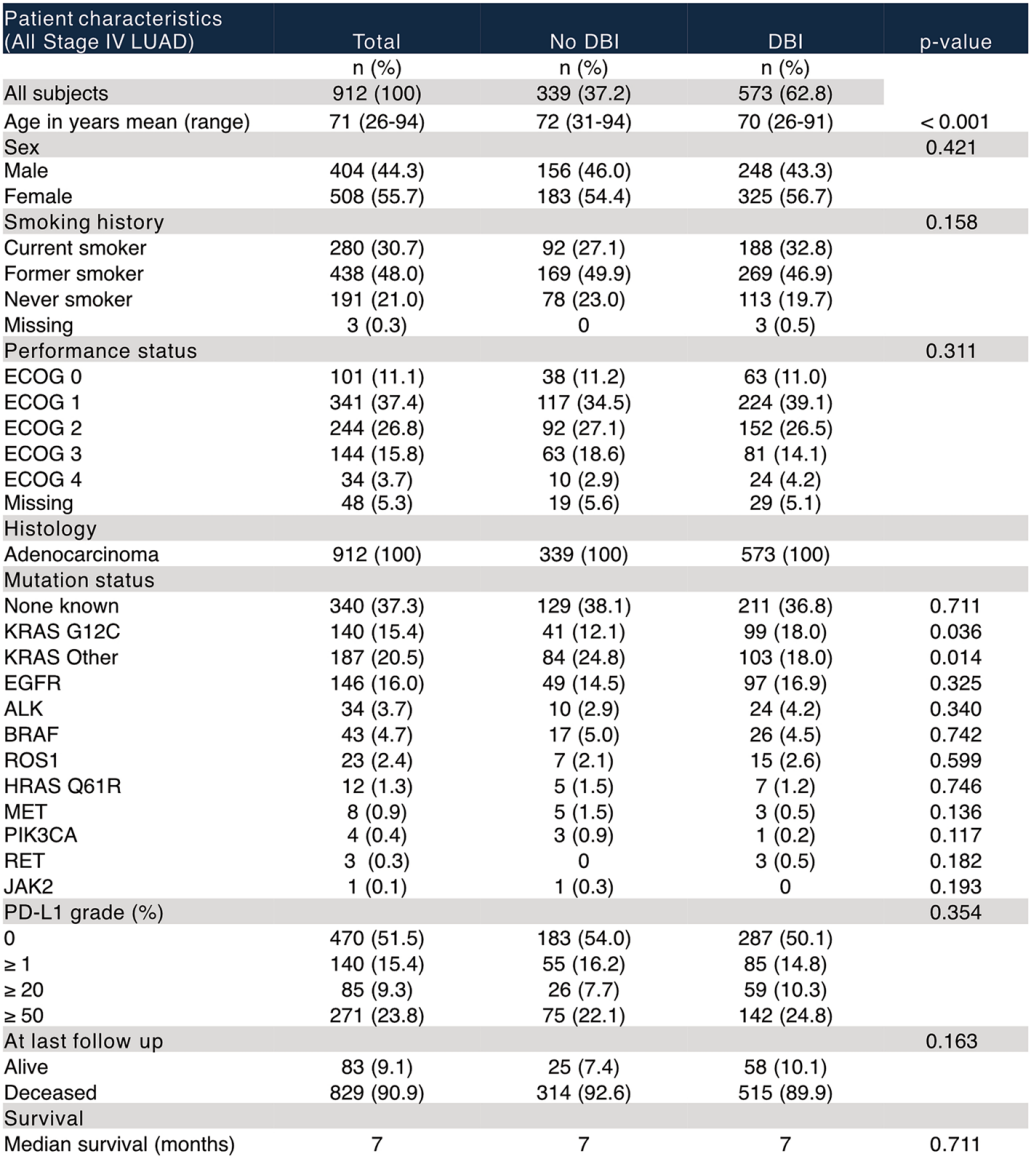
Patient characteristics of entire study population - stratified by presence of diagnostic brain imaging (DBI)

### Study objectives

The primary outcome of this study was the frequency of DBI, neurological symptoms, presence of BM and OS, defined as the interval between the date of diagnostic sample collection of primary tumors and the date of death from any cause. Patients alive or lost to follow-up were censored at the cut-off date or last contact. Median follow-up time was 35 months (95% CI 31.1–38.9) and was estimated using the reverse Kaplan–Meier method. BM diagnosed within 8 weeks from date of diagnostic sample collection was considered as diagnosed at baseline. The data collection cut-off date was 2024-09-17.

### Statistical analysis

Clinical characteristics were summarized using descriptive statistics and evaluated with univariate analysis in table form. Independent T-test and Pearson’s Chi-square test were used to identify differences in characteristics between groups. Survival was estimated using the Kaplan–Meier method. A log-rank test was used to assess significant differences in OS between groups. Statistical significance was set at *p* < 0.05, and no adjustments were made for multiple comparisons. Data analysis was conducted using IBM SPSS Statistics version 27 and R version 3.4.

## Results

### Patient characteristics

**Fig. 2:**
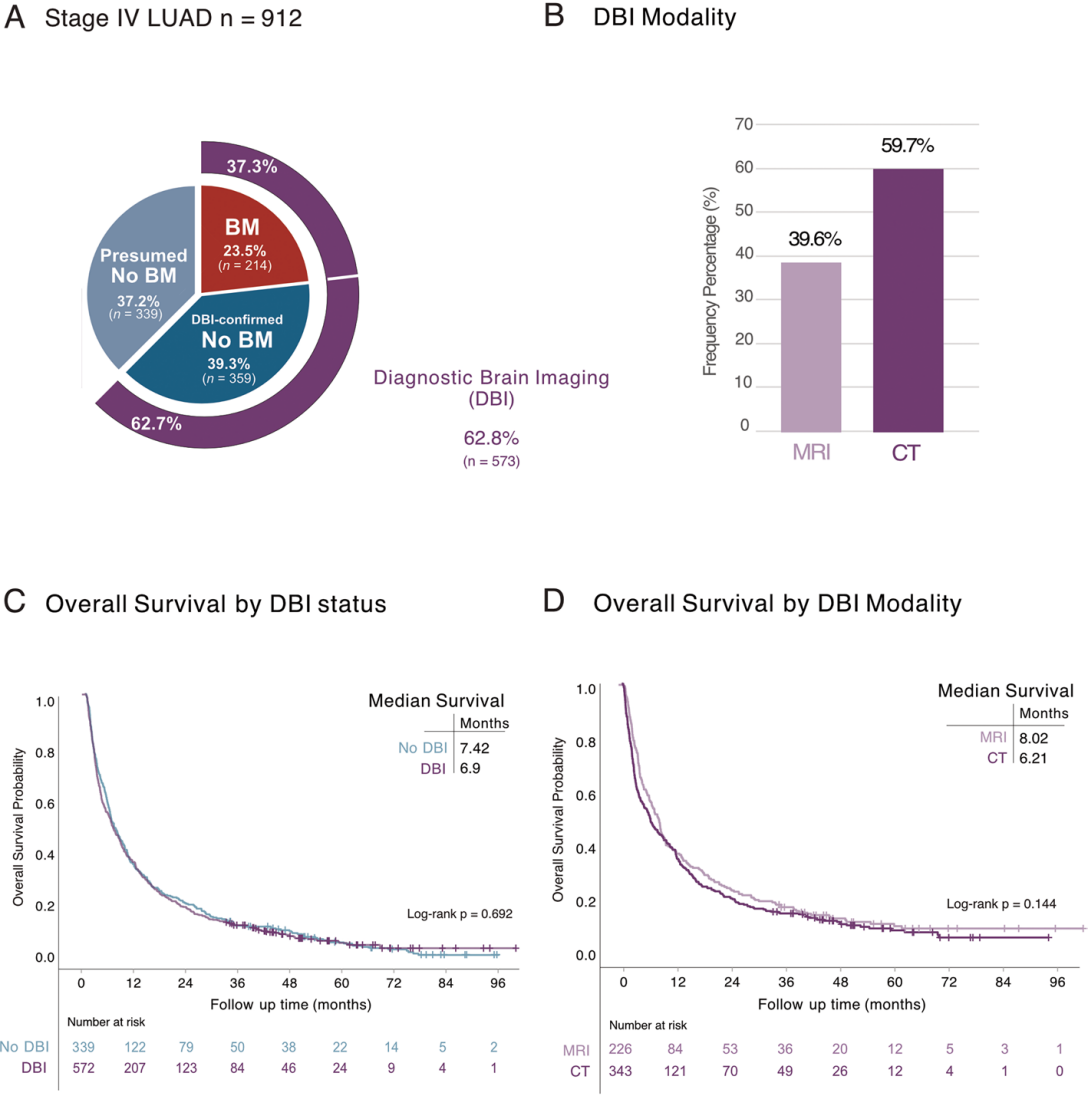
Diagnostic brain imaging (DBI) among patients with stage IV LUAD. **A** Frequency distribution of DBI and brain metastasis (BM) in the study population (*n* = 912). **B** Frequency of CT or MRI as modality of DBI. **C** Kaplan-Meier estimates comparing OS stratified by presence (purple) or absence (blue) of DBI. **D** Kaplan-Meier estimates comparing OS stratified by CT (dark purple) or MRI (light purple) as modality of DBI

Among all stage IV LUAD patients, the majority (*n* = 573, 63%) received DBI (Table 1), and 23% (37% of group that received DBI) had BM (Supplementary Tables 1 and Fig. 2A). There were no significant differences in patient characteristics between the groups except that patients who had a DBI were slightly younger with a mean age of 70 years (range 26–91 years) than those who did not (72 years (31–94) (*p* = < 0.001) (Table 1). Among all who received DBI, 60% received CT and 40% received MRI (Fig. 2B). Importantly, there was no significant difference in OS between the groups that received DBI and those who did not (log-rank *p =* 0.692) (Fig. 2C) and the DBI modality did not impact OS (log-rank *p =* 0.144) (Fig. 2D).

**Table 2:**
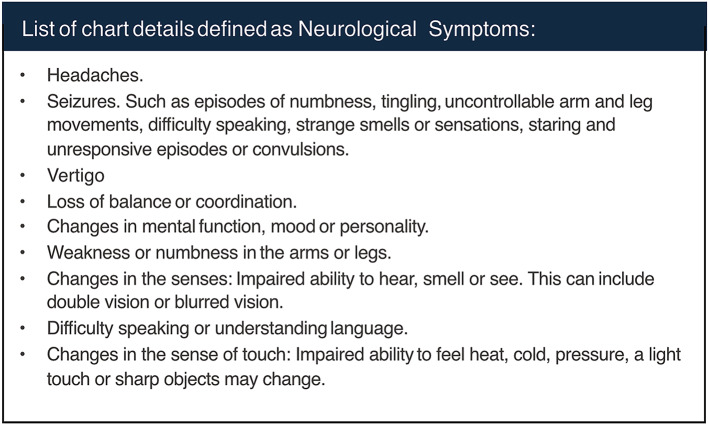
List of chart details defined as neurological symptoms

### DBI findings in BM patients

**Fig. 3:**
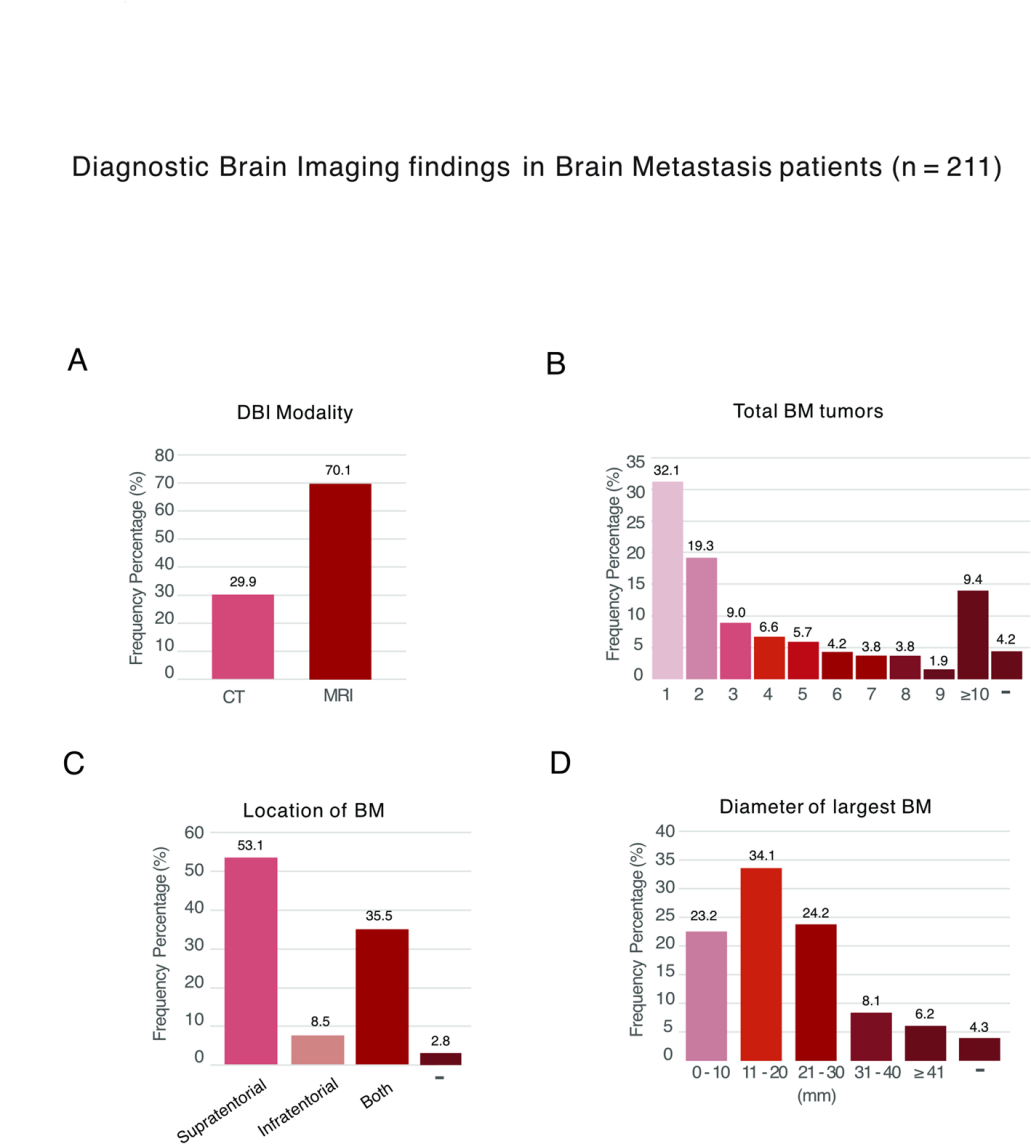
DBI findings in stage IV LUAD patients with BM. (-) Not specified in charts or DBI report for (**B**) Number, (**C**) Location, or (**D**) Numerical size measurement

The majority (70%) of BM was diagnosed with MRI, while around a third (29.9%) received only CT (Fig. 3A). At diagnosis, 32% of BM patients had 1 BM, with fewer patients presenting with increasing numbers of BM up to 9 tumors. The proportion of patients with more than 10 BM at diagnosis was 9% (Fig. 3B). The most common location of BM was supratentorial (53%) followed by having both supra- and infratentorial BM (36%) and only infratentorial BM was rare (9%) (Fig. 3C). When looking at the size of the largest BM at diagnosis, 34% of all BM on DBI were 11–20 mm, and 60% BM were less than 30 mm in diameter (Fig. 3D).

### Neurological symptoms among stage IV LUAD with DBI

**Fig. 4:**
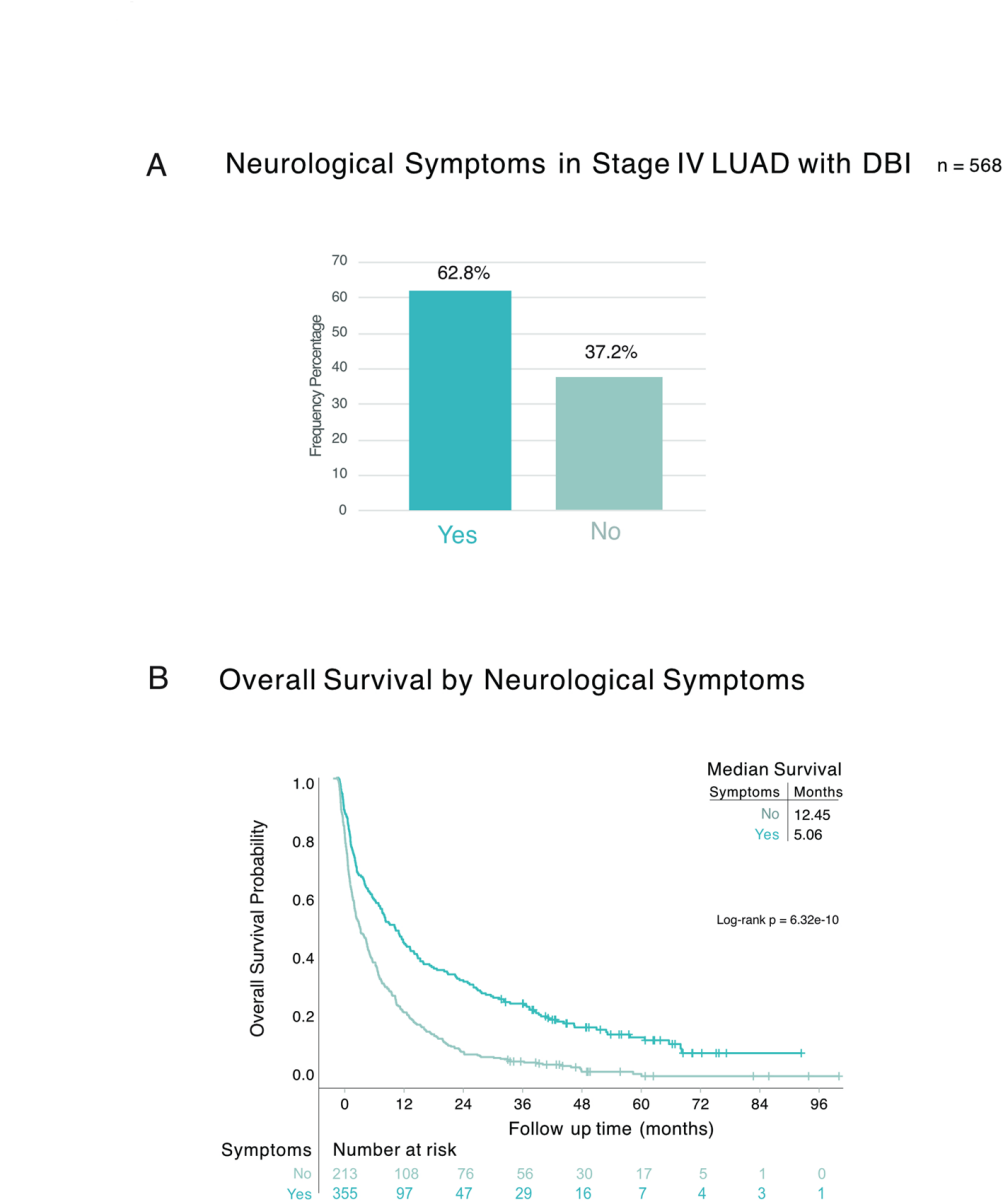
Neurological symptoms in stage IV LUAD with DBI. **A** Frequency of presence (Yes) or absence (No) of neurological symptoms in the study population (*n* = 568). **B** Kaplan-Meier estimates comparing OS stratified by presence (Yes) or absence (No) of neurological symptoms

Next, we investigated whether the DBI among stage IV LUAD patients were received due to the presence of neurological symptoms. We found that the majority (63%) of DBI referrals were due to neurological symptoms (Table 3; Fig. 4A). The group with symptoms had higher mean age of 71 (29–91) years compared with 67 (26–88) years in asymptomatic patients (*p* < 0.001) and had a worse performance status with the majority being ECOG ≥ 2 (56% of symptomatic patients vs. 39% of asymptomatic patients) (*p* = 0.005) (Table 3). Additionally, patients with neurological symptoms had significantly worse OS with a median survival of 5 compared to 12 months for those without symptoms (log-rank *p* < 0.0001) (Fig. 4B).

**Table 3:**
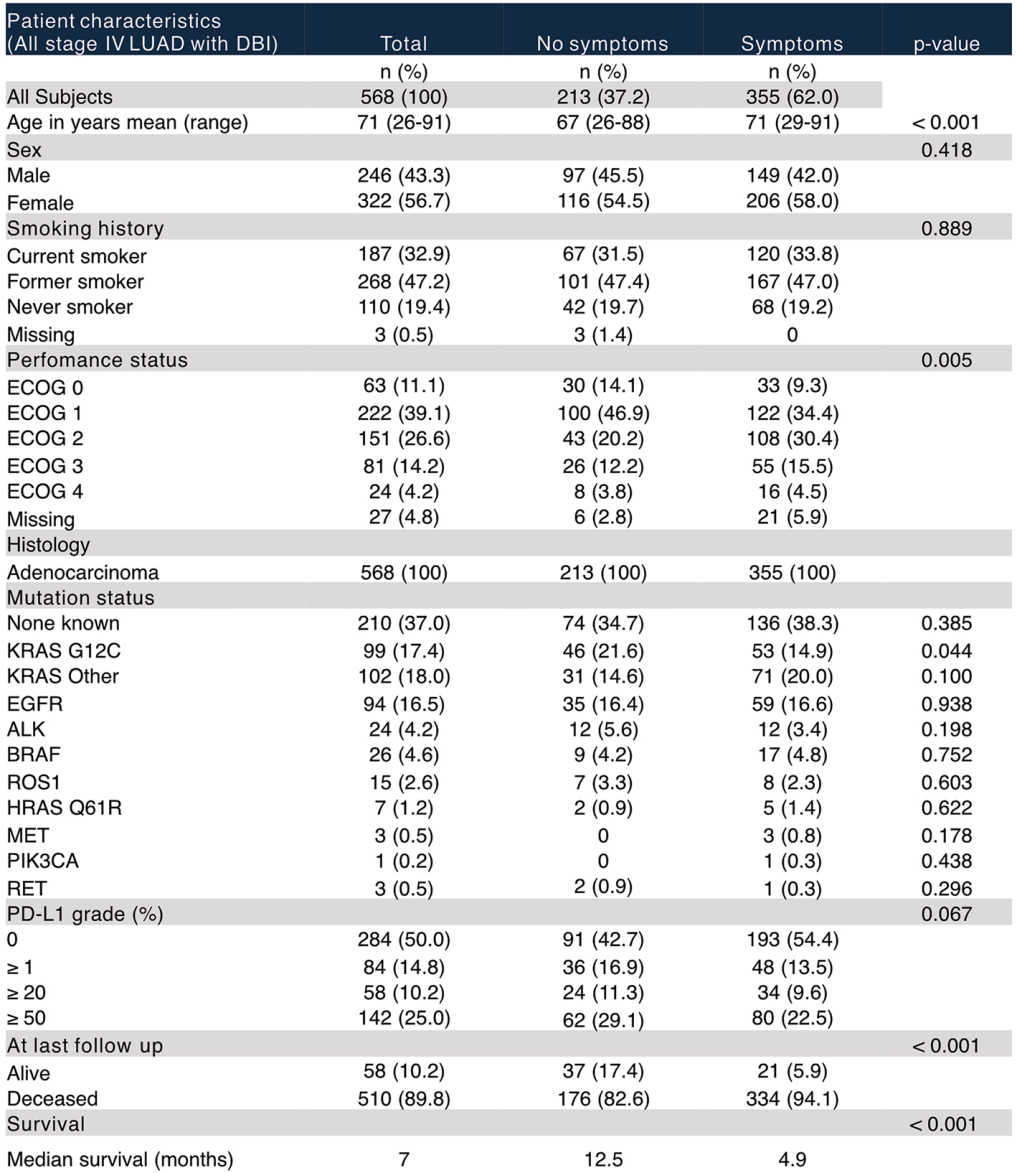
Patient characteristics of all stage IV LUAD patients with DBI - stratified by presence or absence of neurological symptoms

### Neurological symptoms in relation to BM detection at diagnosis in LUAD

**Fig. 5:**
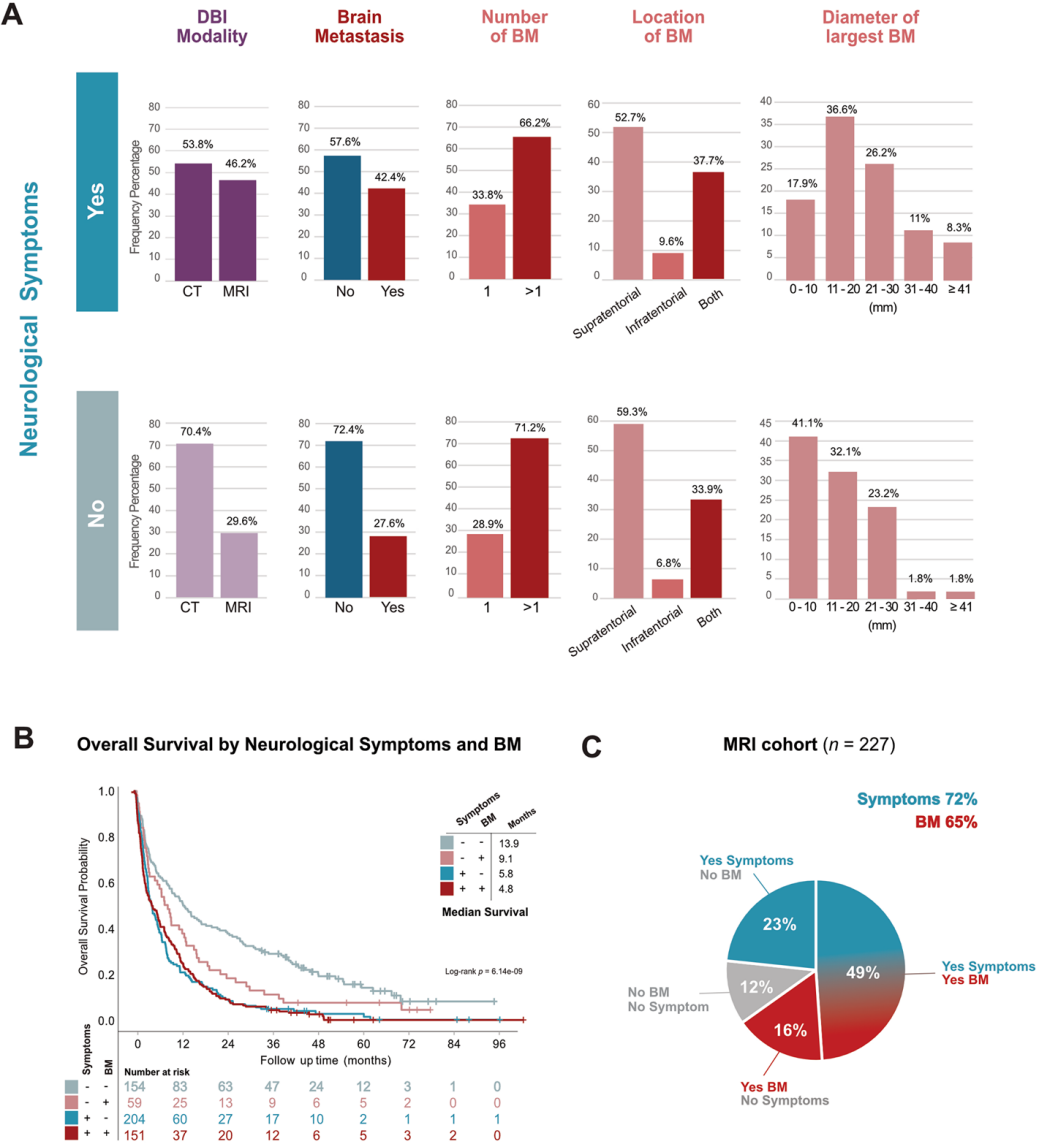
Neurological symptoms in relation to BM in stage IV LUAD with DBI. **A** Frequency distribution of DBI findings in stage IV LUAD in relation to Presence (Yes, top panels) or absence (No, bottom panels) of neurological symptoms: imaging modality (purple), presence of BM (red or blue), and DBI findings in BM patients (shades of red). **B** Kaplan-Meier estimates comparing OS stratified by presence or absence of neurological symptoms and of BM. **C** Frequency distribution of symptoms (light blue) and BM (red) among all patients who received MRI

Next, to probe the value of neurological symptoms as an indicator for diagnostic BM screening, we studied DBI findings in the subgroups with and without symptoms. In the absence of neurological symptoms, 70% received CT and 30% received MRI, while higher proportion (46%) received an MRI when neurological symptoms were present (Fig. 5A).

Importantly, 58% of all stage IV with neurological symptoms at diagnosis had no BM on DBI, while 28% of those who received DBI even in the absence of any neurological symptoms at diagnosis had BM (Fig. 5B).

Distribution of number or location of BM were similar between groups regardless of neurological symptoms. However, when looking at size of the largest BM tumor, we found that while only 18% of BM tumors under 10 mm were detected when DBI was performed in the presence of neurological symptoms, up to 41% BM were detected with tumors less than 10 mm in size when DBI was performed even though no neurological symptoms were present (Fig. 5A).

### Neurological symptoms impact OS independent of BM

Further we investigated the impact of the presence of neurological symptoms in relation to whether a BM was detected with DBI. As expected, patients without symptoms and no BM had a longer median OS of 14 months (Fig. 5B). However, the patients presenting symptoms without BM had almost as short median survival as the patients with symptoms and BM, with a median of 5.8 vs. 4.8 months (log-rank *p =* 1.00) (Fig. 5B and Supplementary Table 2). The patients without neurological symptoms and BM had a numerically better OS than the patients with symptoms and BM with a median of 9.1 months vs. 4.8 months (*p* = 0.253) (Fig. 5B and Supplementary Table 2).

### Characteristics of the MRI cohort

As the superior sensitivity of MRI implies the most accurate reflection of true prevalence of BM, we investigated the relationship between neurological symptoms and BM presence in all stage IV patients who received MRI. We found that among all patients who received screening MRI at diagnosis, 72% had neurological symptoms and 65% had BM (Fig. 5C). While 23% had neurological symptoms in the absence of BM, 49% had both symptoms and BM. Importantly, among patients who received MRI in the absence of neurological symptoms, 57% had asymptomatic BM.

### MRI detects BM missed by CT

Among all patients who received MRI, 59% received a CT before the MRI (*n* = 133) and within this group 87% (*n* = 116) were positive for BM (Table 4). In 8 of these cases (7%), CT did not detect the BM that MRI subsequently revealed. All 8 patients had neurological symptoms at diagnosis.

**Table 4:**
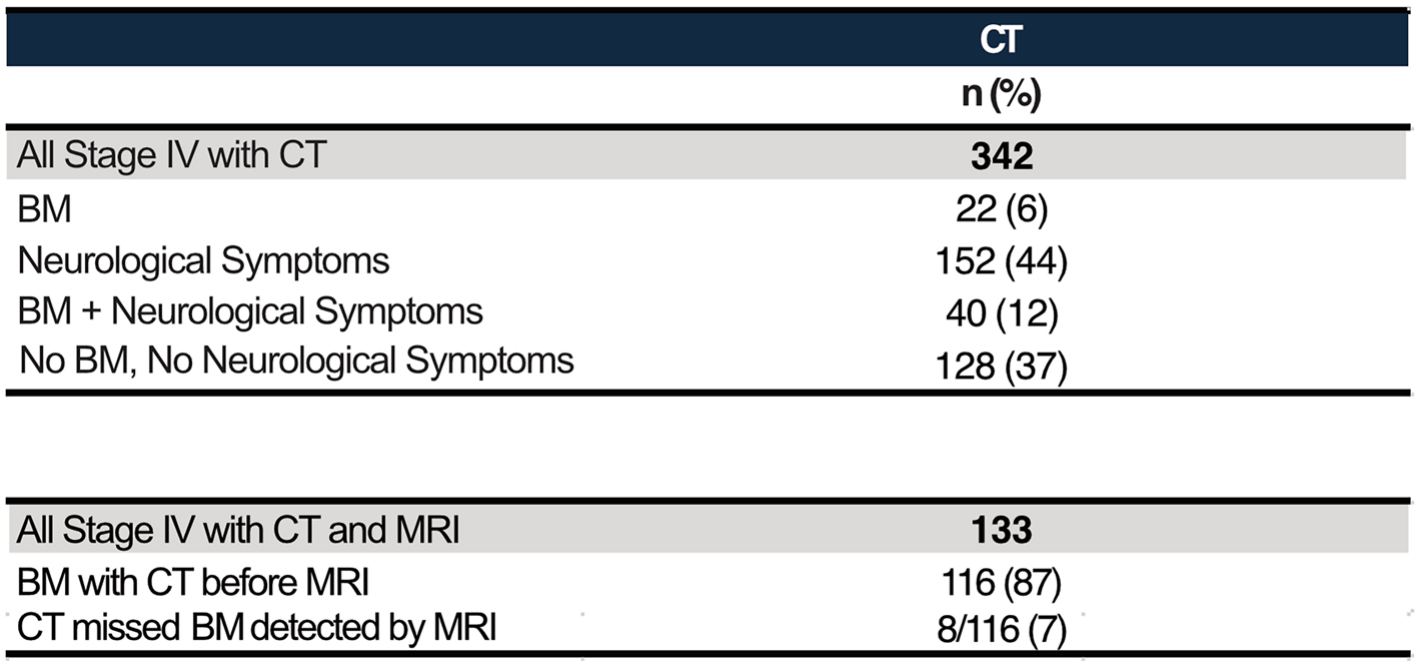
Distribution of BM and neurological symptoms among stage IV patients imaged with CT

## Discussion

Current guidelines recommend brain imaging at diagnosis for all patients with metastatic lung cancer and mandate it for those with neurological symptoms or signs [14, 16, 17, 20, 21]. This real-world multi-center study investigated how DBI is used in relation to neurological symptoms in patients with newly diagnosed stage IV LUAD. While nearly two-third of patients received DBI, a relatively large proportion did so in the absence of neurological symptoms. Importantly, our findings demonstrate that relying solely on neurological symptoms to guide imaging decisions risks under-detecting asymptomatic BM, which were found in nearly one-third of asymptomatic patients who received imaging. The observed age difference between patients who received DBI and those who did not suggests a potential selection bias in the clinical decision making to pursue DBI, possibly reflecting age-related considerations in treatment aggressiveness or perceived prognosis.

Our findings raise the possibility that asymptomatic patients with BM may be underdiagnosed when imaging is symptom-triggered. The detection of smaller BMs in asymptomatic patients suggests that earlier identification may be feasible with routine imaging, potentially enabling broader treatment options before symptom onset. However, while DBI might allow for earlier detection, our study did not find a corresponding improvement in OS based on DBI presence or modality. This lack of association may reflect uniform treatment approaches regardless of imaging timing or modality, or it may indicate that interventions triggered by early detection are not yet sufficient to impact survival outcomes. In this study we did not evaluate post-diagnostic treatment strategies, which limits our ability to determine whether detection of brain metastases—particularly in asymptomatic patients—led to meaningful changes in clinical management or improved outcomes. This represents an important limitation in interpreting the prognostic relevance of early BM detection in this cohort. While our findings raise concerns that symptom-triggered imaging may miss asymptomatic brain metastases, they do not demonstrate a clinical benefit of routine imaging and should be interpreted in the context of broader considerations such as treatment strategy, prognosis, and resource allocation.

Our findings align with and extend contemporary guidance and syntheses indicating that neurological symptoms alone are an unreliable trigger for baseline brain imaging in advanced NSCLC. Multiple consensus/guideline documents now emphasize MRI as the preferred modality and acknowledge high rates of occult, asymptomatic brain metastases at presentation in stage III–IV disease [14, 16, 17, 20, 21]. Systematic and structured reviews likewise conclude that routine baseline imaging with MRI detects otherwise occult lesions and can alter initial management (local therapy planning, systemic regimen selection, and trial eligibility) [12, 15, 30]. Real-world and health-system studies further report that baseline MRI changes intent or modality of therapy and may yield signals toward improved care quality in fitter patients, supporting a lower threshold for MRI even in the absence of symptoms [19, 26]. Against this backdrop, our regional cohort demonstrates that 28% of asymptomatic patients who were imaged at all (and importantly, 57% of asymptomatic patients who received MRI) harbored BM, and that symptom-triggered imaging preferentially identifies larger lesions, aligning with prior observations that symptom-detected BM are larger and clinically more disruptive at presentation [27, 28]. Together, these data highlight the gap between symptom-based recommendations and real-world disease biology, and support consideration of routine baseline brain MRI at stage IV diagnosis.

Importantly in our cohort, stage IV patients with neurological symptoms had worse OS regardless of the presence of BM. This group was systematically different from those without symptoms, with increased age and worse PS. These findings underscore the need for comprehensive clinical assessment beyond imaging results, as symptoms may also stem from comorbidities such as leptomeningeal disease, para-neoplastic syndromes, and shorter OS could be due to treatment-associated toxicity, factors that were not assessed in this study. Also, there are probably several BM patients hidden in this group, for example, those with CT only. This patient group should be further characterized in future studies to investigate BM-independent mechanisms of neurological symptoms associated with worse prognosis in stage IV LUAD.

Our results also show a preference for CT over MRI in asymptomatic patients. This preference may contribute to under detection of BM and reflects real-world variability in imaging access and clinical practice when imaging is not mandatory. The choice between CT and MRI was at the clinician’s discretion, with MRI often reserved for cases with inconclusive CT findings or more severe symptoms. This heterogeneity in imaging modality may have influenced BM detection rates. While current ESMO recommendations favor MRI, limited availability or contraindications may explain reliance on CT in our cohort.

Interpretation of the CT-screened cohort must be framed by CT’s known sensitivity limits. In our subset with CT prior to MRI, among patients ultimately MRI-positive, 7% had a preceding CT that was negative, and all eight of these CT-negative/MRI-positive cases were symptomatic at presentation. Thus, a negative CT should not be considered definitive, particularly in symptomatic patients, as CT-only strategies risk false negatives that can delay stereotactic/radiation planning or constrain systemic options [12, 13, 16, 17, 20, 25]. Importantly, the absence of BM in some patients with neurological symptoms further underscores the known limitations of CT in comparison to MRI. Where MRI access or contraindications drive initial CT use, negative CT findings should be interpreted with caution, and MRI should be obtained whenever possible [12, 13, 16, 17, 20, 25].

In the MRI-screened cohort, which best reflects true statistics given MRI’s superior sensitivity, brain metastases were present in 65% of patients. Although 72% of MRI recipients had neurological symptoms, 57% of the asymptomatic patients who underwent MRI also had BM—showing that symptoms alone miss a substantial fraction of disease at baseline. These findings are directionally concordant with guideline and structured-review conclusions that MRI detects clinically relevant, small-volume or posterior fossa disease that CT may miss and therefore should be prioritized for baseline staging in stage IV NSCLC when feasible [12, 13, 15–17, 20, 29]. Clinically, the high yield in asymptomatic patients supports early MRI because it can affect local therapy planning, systemic regimen selection, and trial eligibility [15–17, 25, 29].

While EGFR and ALK-mutated stage IV NSCLC now receive routine DBI, our dataset still showed comparable number of patients with these mutations with the group that did not receive DBI. Additionally, there was no enrichment of EGFR or ALK even in the patients receiving a DBI without symptoms. This is likely due to guidelines requiring DBI for all EGFR and ALK-mutated stage IV were updated during the study period.

Recent systematic reviews have investigated the diagnostic and clinical implications of detecting brain metastases in NSCLC patients, particularly those without neurological symptoms. Mayer et al. [15] conducted a systematic review of brain imaging practices in NSCLC staging and concluded that routine imaging—especially in stage III–IV disease—can improve treatment planning by identifying otherwise occult BM. Chakraborty et al. [12] performed a systematic review examining the impact of imaging modality and timing on BM detection in NSCLC, and reported that the use of MRI at diagnosis is associated with earlier BM detection and more effective therapeutic decision-making. Both reviews underscore the clinical rationale for broader brain imaging strategies but note a lack of real-world data on how symptom status currently influences imaging use. Our study addresses this by analyzing DBI practices in stage IV LUAD, where in contrast to stage I-III disease, brain imaging is not standardized and recommended to be guided by symptoms.

Compared to the prior primary studies discussed in this context, our methodology is distinct. Dubbé-Pelletier et al. [22] and Waizman et al. [29] assessed BM prevalence or outcomes in strictly asymptomatic patients undergoing routine MRI—excluding symptomatic patients and not reflecting routine clinical decision-making. Kim et al. [23] included all NSCLC patients but only included patients with DBI, and did not take symptomatology into account. Ohhara et al. [25] and Steindl et al. [31] focused only on patients with established BM and found that those presenting with symptoms had poorer survival, but these studies did not evaluate how imaging decisions were made. Farris et al. [27] further showed that symptom-detected BM were larger, more likely to require hospitalization, and less amenable to focused therapy than screen-detected lesions—underscoring the consequences of symptom-based delays. Importantly, none of these studies assessed imaging patterns across all patients at the time of stage IV diagnosis, the only stage where DBI recommendation is ambiguous. In contrast, our study captures the full range of stage IV LUAD patients—both with and without symptoms and with and without BM—and evaluates how symptom presentation influences DBI use, modality, detection rates, and survival in stage IV, the only stage where DBI recommendation in NSCLC remains ambiguous. This broader design enables us to assess the limitations of symptom-triggered imaging strategies in this group and offers complementary evidence to existing literature which predominantly focuses either on outcomes post-diagnosis or controlled screening cohorts.

Given our results, new treatment strategies like SRS of several BMs, newer systemic therapies with improved blood-brain barrier penetrance, and as screening is already routine for liver and adrenal metastases in this group, routine brain imaging at diagnosis with MRI, regardless of the presence of neurological symptoms, is likely warranted for all patients with metastatic lung cancer.

### Limitations

This study has several important limitations. First, the retrospective design inherently introduces a risk of incomplete or inconsistent documentation, particularly regarding neurological symptoms and the rationale for imaging decisions. The classification of symptoms relied on chart review and clinician judgment, which may vary across sites and cases. Future prospective studies should incorporate standardized and validated symptom assessment tools to improve the consistency and accuracy of neurological symptom classification. Second, the study does not account for potential confounding factors such as comorbidities, disease burden at other metastatic sites, or systemic treatment decisions, which may independently influence neurological symptoms and OS. Symptoms may also stem from comorbidities such as leptomeningeal disease, paraneoplastic syndromes, metabolic disturbances, or treatment toxicity. These factors were not assessed in our study and may contribute to poorer outcomes in patients with symptoms but no BM. Third, the selection of patients for DBI was not standardized, and younger or fitter patients were more likely to undergo imaging, suggesting potential selection bias. The choice between CT and MRI was at the clinician’s discretion, with MRI often reserved for cases with inconclusive CT findings or more severe symptoms. This heterogeneity in imaging modality may have influenced BM detection rates.

Furthermore, in cases with multiple brain metastases, we recorded only the largest lesion due to retrospective data constraints, which may underestimate intracranial disease burden. Importantly, because DBI was not routinely performed in all patients, we cannot estimate the true prevalence of asymptomatic BM, nor assess how BM detection influenced clinical decision-making or treatment outcomes. As such, our findings raise concerns about under-detection when imaging is limited to symptomatic patients in Stage IV disease, and prospective studies with standardized imaging protocols and treatment documentation are needed to validate these results.

## Conclusion

Neurological symptoms alone are an unreliable indicator for detecting brain metastases in stage IV lung adenocarcinoma. In our comprehensive regional-level cohort from western Sweden, a notable proportion of asymptomatic patients who underwent brain imaging were found to have BM, suggesting that symptom-based screening may miss clinically relevant cases.

Our retrospective design limits conclusions about how symptom status affects BM detection rates, treatment decisions, or prognostic outcomes. Prospective studies are needed to clarify these relationships and inform future screening strategies.

## Supplementary Information

Supplementary material 1.

## Data Availability

The datasets used and/or analyzed during the current study is available from the corresponding author on reasonable request.
