# Supplementary material for: Brain metastasis in stage IV lung adenocarcinoma is frequently missed by symptom-based screening
Source: Discov Oncol. 2025 Dec 11;17:95. doi: 10.1007/s12672-025-04210-7 (PMC12804454; doi:10.1007/s12672-025-04210-7)
Supplement: Supplementary file 1 — Supplementary material 1. [file 12672_2025_4210_MOESM1_ESM.pdf]

Supplementary Table 1

Patient characteristics (All stage IV LUAD with DBI)	Total	No BM	BM	*p*-value
	n (%)	n (%)	n (%)	
All subjects	573 (62.8)	359 (62.7)	214 (37.3)	
Age in years mean (range)	71 (26-91)	70 (26-91)	69 (34-87)	0.041
**Sex**				0.043
Male	248 (43.3)	167 (46.5)	81 (37.9)	
Female	325 (56.7)	192 (53.5)	133 (62.1)	
**Smoking history**				0.461
Current smoker	188 (32.8)	111 (30.9)	77 (36.0)	
Former smoker	269 (46.9)	173 (48.2)	96 (44.9)	
Never smoker	113 (19.7)	73 (20.3)	40 (18.7)	
Missing	3 (0.5)	2 (0.6)	1 (0.5)	
**Perfomance status**				0.469
ECOG 0	63 (11.0)	36 (10.0)	27 (12.6)	
ECOG 1	224 (39.1)	141 (39.3)	83 (38.8)	
ECOG 2	152 (26.5)	91 (25.3)	61 (28.5)	
ECOG 3	81 (14.1)	57 (15.9)	24 (11.2)	
ECOG 4	24 (4.2)	16 (4.5)	8 (3.7)	
Missing	29 (5.1)	18 (5.0)	11 (5.1)	
**Histology**				
Adenocarcinoma	573 (100)	359 (100)	214 (100)	
**Mutation status**				
None known	211 (36.8)	133 (37.0)	78 (36.4)	0.886
KRAS G12C	99 (18.0)	65 (18.1)	34 (15.9)	0.497
KRAS Other	103 (18.0)	69 (19.2)	34 (15.9)	0.315
EGFR	97 (16.9)	48 (13.4)	49 (22.9)	0.003
ALK	24 (4.2)	15 (4.2)	9 (4.2)	0.987
BRAF	26 (4.5)	21 (5.8)	5 (2.3)	0.051
ROS1	15 (2.6)	14 (3.9)	1 (0.5)	0.013
HRAS Q61R	7 (1.2)	3 (0.8)	4 (1.9)	0.276
MET	3 (0.5)	1 (0.3)	2 (0.9)	0.293
PIK3CA	1 (0.2)	0	1 (0.5)	0.195
RET	3 (0.5)	3 (0.8)	0	0.180
**PD-L1 grade (%)**				0.341
0	287 (50.1)	170 (47.4)	117 (54.7)	
≥ 1	85 (14.8)	59 (16.4)	26 (12.1)	
≥ 20	59 (10.3)	39 (10.9)	20 (9.3)	
≥ 50	142 (24.8)	91 (25.3)	51 (23.8)	
**At last follow up**				0.105
Alive	58 (10.1)	42 (11.7)	16 (7.5)	
Deceased	515 (89.9)	317 (88.3)	198 (92.5)	
**Survival**				0.038
Median survival (months)	7	8	6	

Supplementary Table 1 Patient characteristics of all stage IV LUAD patients with DBI - stratified by presence or absence of brain metastasis (BM). p-values are from Pearson's Xi

Supplementary Table 2

Comparison groups	p-value
· No Symptoms & BM vs No Symptoms & No BM	0.262
· Symptoms & No BM vs No Symptoms & No BM	2.36E-08
· Symptoms & BM vs No Symptoms & No BM	8.45E-07
· Symptoms & No BM vs No Symptoms & BM	0.198
· Symptoms & BM vs No Symptoms & BM	0.253
· Symptoms & BM vs Symptoms & No BM	1

Supplementary Table 2 Pairwise Log-Rank Test p-values for comparison of OS groups in Figure 5B.
